# Supplementary material for: Intimal Sarcoma with MDM2/CDK4 Amplification and p16 Overexpression: A Review of Histological Features in Primary Tumor and Xenograft, with Immunophenotype and Molecular Profiling
Source: Int J Mol Sci. 2023 Apr 19;24(8):7535. doi: 10.3390/ijms24087535 (PMC10141691; doi:10.3390/ijms24087535)
Supplement: Supplementary file 1 [file ijms-24-07535-s001.zip › ijms-2306342-supplementary.pdf]

Supplementary Table S1a. Immunohistochemistry analysis. Antibodies, sources, clone, dilutions, pretreatments and staining patterns (1)

ANTIBODIES	SOURCE	CLONE	DILUTION	PRETREATMENT	STAINING PATTERN
**MDM2**	DAKO	12E7	prediluted	PTLINK high pH	nuclear
**CDK4**	SANTA CRUZ	C-22	1/50	PTLINK high pH	nuclear
**Pan-TRK**	SANTA CRUZ	C14	1/100	PTLINK high pH	cytoplasmic
**H-TERT**	ABCAM	2C4	1/50	PTLINK low pH	nuclear
**KI67**	DAKO	MIB-1	prediluted	PTLINK low pH	nuclear
**FLI-1**	SANTA CRUZ BIOTHECNOLOGY	H60	1/200	PTLINK high pH	nuclear
**CD31**	DAKO	JC70A	prediluted	PTLINK high pH	cytoplasmic
**CD34**	DAKO	QBend10	prediluted	PTLINK high pH	cytoplasmic/membranous
**CD117**	DAKO	polyclonal	1/100	PTLINK high pH	cytoplasmic/membranous
**SMA**	DAKO	1A4	prediluted	PTLINK high pH	cytoplasmic
**DESMIN**	DAKO	D33	prediluted	PTLINK high pH	cytoplasmic
**H-caldesmon**	DAKO	h-CD	prediluted	PTLINK high pH	cytoplasmic
**Factor VIII**	DAKO	polyclonal	prediluted	PTLINK high pH	cytoplasmic
**D2.40 (podoplanin)**	DAKO	D2-40	prediluted	PTLINK high pH	cytoplasmic
**C-MYC**	DAKO	EP121	prediluted	PTLINK high pH	nuclear

Supplementary Table S1b. Immunohistochemistry analysis. Antibodies, sources, clone, dilutions, pretreatments and staining patterns (2)

ANTIBODIES	SOURCE	CLONE	DILUTION	PRETREATMENT	STAINING PATTERN
**EMA**	DAKO	E29	prediluted	PTLINK high pH	cytoplasmic
**MyoD1**	DAKO	5.8A	1/50	PTLINK high pH	nuclear
**p53**	DAKO	DO-7	prediluted	PTLINK high pH	nuclear
**S100**	DAKO	polyclonal	prediluted	PTLINK high pH	cytoplasmic/nuclear
**FOS-B**	CELL SIGNALING	5G4	1/100	PTLINK low pH	nuclear
**EGFR**	DAKO	H11	1/50	PTLINK high pH	membranous/cytoplasmic
**PDGFRA**	NEOMARKERS	polyclonal	1/100	PTLINK high pH	cytoplasmic
**GLI-1**	SANTA CRUZ	A7	1/500	PTLINK low pH	nuclear
**STAT-6**	Gennova	EP325	1/25	PTLING high pH	nuclear
